# Supplementary material for: The interactome of intact mitochondria by cross-linking mass spectrometry provides evidence for coexisting respiratory supercomplexes
Source: Mol Cell Proteomics. 2017 Dec 8;17(2):216–32. doi: 10.1074/mcp.RA117.000470 (PMC5795388; doi:10.1074/mcp.RA117.000470)
Supplement: Supplemental Data [file supp_RA117.000470_133922_0_supp_23978_fzffwf.zip › spectra_annotation/mito_spectra_annotation/292-1-4-1-6-1.pdf]

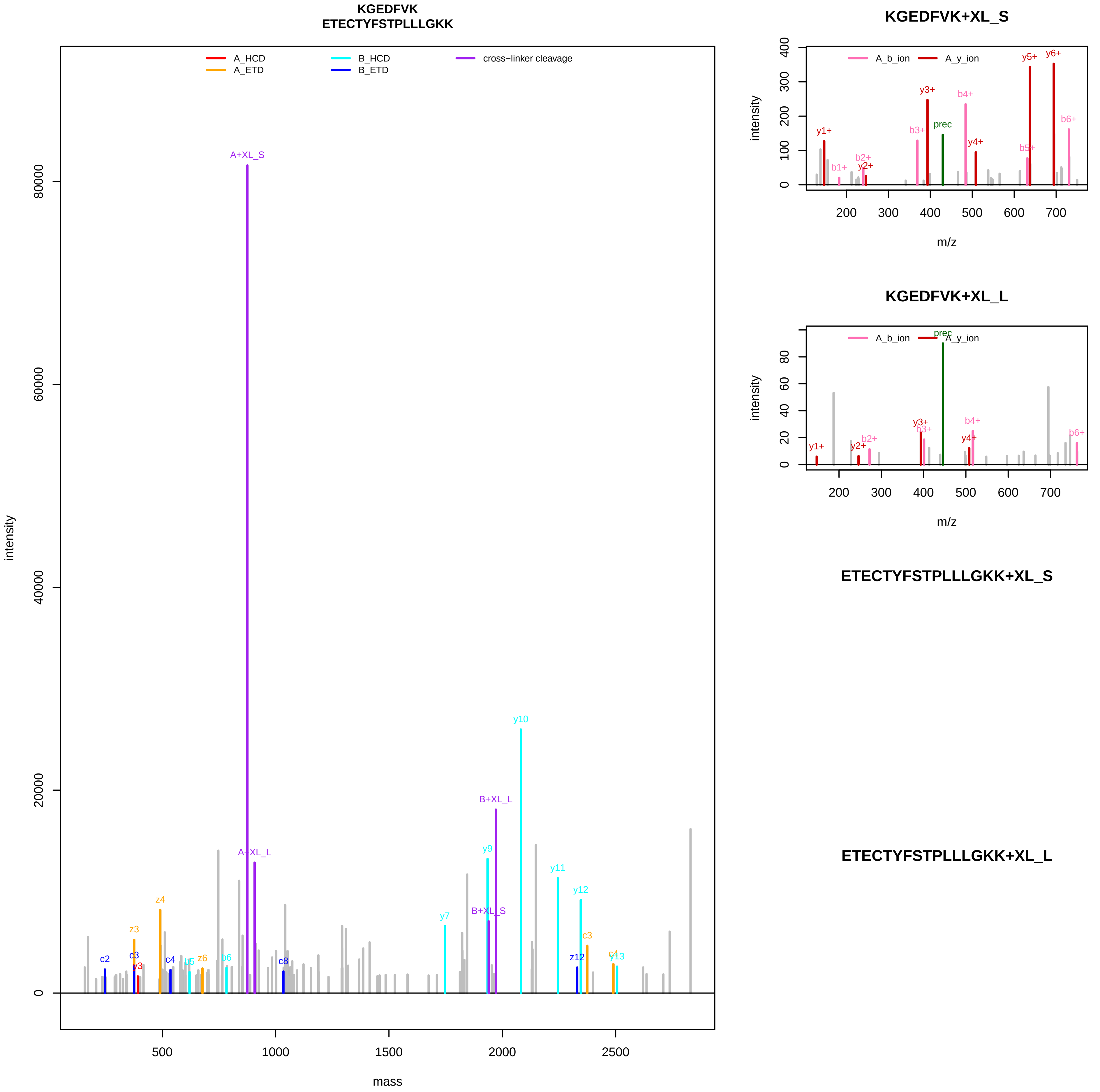
KGEDFVK
ETECTYFSTPLLLGKK
A_HCD
A_ETD
B_HCD
B_ETD
cross−linker cleavage
A+XL_S
A+XL_L
B+XL_L
B+XL_S
y10
y9
y7
y11
y12
y13
z3
z4
z6
z12
c2
c3
c4
c8
y3
b5
b6
c3
c4
intensity
mass
0
20000
40000
60000
80000
500
1000
1500
2000
2500
KGEDFVK+XL_S
A_b_ion
A_y_ion
y1+
b1+
b2+
y2+
b3+
y3+
prec
b4+
y4+
b5+
y5+
y6+
b6+
m/z
KGEDFVK+XL_L
ETECTYFSTPLLLGKK+XL_S
ETECTYFSTPLLLGKK+XL_L
